# Mechanisms of severe acute respiratory syndrome coronavirus-2 induced liver damage and alteration of some liver biomarkers: A review

**DOI:** 10.1097/MD.0000000000033517

**Published:** 2023-05-12

**Authors:** Fikadu Seyoum

**Affiliations:** a Department of Medical Biochemistry, College of Medicine and Health Sciences, Ambo University, Addis Ababa, Ethiopia.

**Keywords:** liver injury, liver function tests, SARS-CoV-2

## Abstract

The most serious problem for people suffering from severe acute respiratory syndrome coronavirus-2 (SARS-CoV-2) infection is liver damage. The liver is a frequently affected organ due to the metabolizing and detoxifying functions of several endogenous and exogenous molecules. In COVID-19-affected individuals, even moderate loss of hepatic function could dramatically affect the therapeutic efficacy of antiviral drugs metabolized in the liver. The clear mechanism of hepatocellular damage from SARS-CoV-2 infection is not fully understood. The main objective of this review is to identify potential mechanisms of SARS-2 induced liver damage, treatment outcomes in SARS-CoV-2-infected patients, and future direction. Electronic databases including Web of Science, Google Scholar, MEDLINE, Scopus, and Cochrane library were used to systematically search without limitation of publication date and status. Observational, retrospective cohort, prospective case-control, cohort studies, cross-sectional studies, or clinical trials were included. Liver damage in coronavirus patients is characterized by histopathological changes and abnormal elevation of some liver function tests. These abnormalities include elevation of Alanine aminotransferase, Aspartate aminotransferase, Gamma-glutamyl transferase, Alkaline phosphatase, and Serum bilirubin levels. Histopathological changes of the liver might consist of complete or partial thrombosis of the portal and sinusoidal vessels, portal tract fibrosis, and focally markedly enlarged and fibrotic hepatocytes. Understanding the fundamental molecular and immunological processes of COVID-19-related liver injury is essential for the selection of appropriate drugs and the logical development of successful treatment.

## 1. Introduction

In Wuhan, People’s Republic of China, pneumonia of unknown origin was first noticed and reported to the World Health Organization in December 2019. Severe acute respiratory syndrome corona virus-2 (SARS-CoV-2) was the designation given to the illness after it was later determined to be caused by a new coronavirus.^[1]^ Even though most COVID-19 patients suffer from pulmonary symptoms, approximately 5% of individuals suffer from multi-organ dysfunction, which increases of morbidity and mortality from the disease.^[2]^ SARS-CoV-2 is an enveloped virus that can access the cell via angiotensin-converting enzyme 2 (ACE2), the main receptor for this virus. The attachment of SARS-CoV-2 to hepatocyte cells may be mediated by the interaction of the spike (S) protein and host receptor called ACE2. Following attachment of the virus to the host receptor, the S protein is cleaved by the transmembrane serine protease 2, which allows entry of the virus into the host cell.^[3,4]^

The direct effect of SARS-CoV-2 on the liver may not be fully achieved through ACE2 alone. According to some studies, dendritic cell-specific intercellular adhesion molecule-3-grabbing non-integrin and liver-specific intercellular adhesion molecule-3-grabbing non-integrin (L-SIGN) are the alternative receptors that promote ACE2-mediated virus infection of hepatic cells.^[5,6]^ Previous studies have also suggested that the S glycoprotein of SARS-CoV-2 may use another transmembrane protein known as CD147 receptor which is highly expressed in hepatocyte cells.^[7–9]^

Once entered into the host cell, the viral components are translated by the host ribosome into polyproteins (pp1a and pp1b) that further cleave into 16 nonstructural proteins.^[10,11]^ Viral replication also occurs in double-membrane vesicles. Additionally, one of the nonspecific proteins called nsp-6 mediated inhibition of auto phagosome and lysosome expansion to favor viral replication. Then after, newly synthesized viral components assemble to form the nucleocapsid and viral envelope at the Endoplasmic reticulum–Golgi intermediate compartment of the hepatocyte. Finally, mature virions are then released through the exploitation of the host vesicular system.^[12,13]^

## 2. Data source and searching strategy

Electronic databases including Web of Science, Google Scholar, MEDLINE, Scopus, and Cochrane library were used to systematically search without limitation of publication date and status. Observational, retrospective cohort, prospective case-control, cohort studies, cross-sectional studies, and clinical trials were included.

## 3. Ethics and dissemination

The protocol of the review does not require ethical approval because it does not involve humans. This article will be published in a peer-reviewed journal and presented at relevant conferences.

## 4. Mechanisms of SARS-CoV-2 induced liver damage, and alteration of some liver biomarkers

The most serious problem for people suffering from SARS-CoV-2 infection is liver damage. Liver is frequently injured due to the metabolizing and detoxifying functions of several endogenous and exogenous molecules. A COVID-19-associated liver injury can be explained as any liver injury that occurs during the course of the disease or treatment in patients with COVID-19. Liver damage in COVID-19 patient is usually characterized by abnormal liver function tests. Moreover, affected liver cells could impair the therapeutic efficacy of antiviral drugs.^[14–16]^

Additionally, infection with SARS-CoV-2 impairs the function of cholangiocellular tight junction proteins which have a protective role in a normal liver. Morphological impairment of the liver can cause the leakage of toxic bile component into the liver parenchyma and cause liver injury.^[17,18]^ Some laboratory findings following SARS-CoV-2 induced liver damage are elevated liver enzymes like Alanine aminotransferase (ALT), aspartate aminotransferase (AST), Gamma-glutamyl transferase, Alkaline phosphatase (ALP), and Serum total bilirubin levels. Additionally, histopathological changes of the liver might encompass complete or partial thrombosis of portal and sinusoidal vessels, portal tract fibrosis, and fibrotic hepatocytes.

Even though the clear mechanism of liver damage in SARS-CoV-2-infected patients is not fully understood, some proposed mechanisms were due to the direct cytopathogenic effects of the SARS virus, cytokine storms, hypoxic from respiratory failure, and certain drugs used for the management of COVID-19.^[19,20]^ Understanding the basic molecular mechanism and processes of COVID-19-related liver injury is essential for the selection of appropriate drug and rational development of successful treatment.

## 5. Several mechanisms of severe acute respiratory syndrome-induced liver damage

Many recent studies are conducted to know the exact mechanism of liver damage by SARS-CoV-2 virus among COVID-19 patients. Some of the recent findings propose that SARS-CoV-2 enters alveolar epithelial cells and hepatocytes via ACE2 receptors.^[21]^ Single-cell RNA-sequencing analysis found that there is a significant ACE2 expression in cholangiocytes.^[22,23]^ ACE2 receptors in the liver cells are expressed mainly in cholangiocytes. However, this receptor is minimally expressed in hepatocytes, and almost absent in Kupffer cells.^[24,25]^ Following the invasion of liver cell by SARS-CoV-2, at early phase the expression of ACE2 in hepatocytes will increase. Systemic inflammation signals such as type I interferon or Interleukin-6 (IL-6)^[26,27]^ also enhance the expression of ACE2 receptors of hepatocyte cells. This mechanism facilitates the easy accessibility of the virus to liver cells. SARS-CoV-2 invades hepatocytes and cholangiocytes through various classical receptors, including Toll-like receptors, Nod-like receptors, and RIG-I-like receptors.^[28]^ Active replication and virus release by infected cells can lead to proptosis, which is responsible for releasing damage associated molecular patterns. When hepatocytes recognize damage associated molecules, proinflammatory cytokines and chemokines, including IL-6, are released. This process switch-on cellular signaling and cytokine molecules attract professional leukocytes like monocytes, macrophages, and T cells to the site, increasing inflammation.^[29]^ Therefore, IL-6 can act to intensify cytokine response syndrome activation. Furthermore, SARS-CoV-2 will downregulate ACE2 as the disease advances. As a result, angiotensin II accumulates and acts as a proinflammatory cytokine via the AT1R-metalloprotease 17 axis to intensify hepatocyte damage.^[1]^

Overactivation of immune cells and proinflammatory cytokines causes a cytokine storm that exacerbates further hepatocyte damage. In the liver, hepatocellular damage caused by proinflammatory cytokines can trigger Kupffer cell activation. Activated Kupffer cells release proinflammatory cytokines and reactive oxygen species, which can continue hepatocyte injury. Nitric oxide generated by activated neutrophils can lead to peroxynitrite formation, cause mitochondrial damage, and induce liver cell necrosis.^[30–32]^

One of the acute complications in COVID-19 patients is severe respiratory distress that can cause tissue hypoxia. When the oxygen delivery to the liver is compromised, liver ischemic injury will develop. Insufficient oxygen supply to the liver will end up with metabolic disturbances resulting from abnormal lipid accumulation, and adinosine triphosphate depletion that leads to hepatocyte cell death. In reperfusion injury, damaged liver cells release damage associated molecular patterns, which activate the complement cascade, and mitochondrial reactive oxygen species production that causes ultimate reperfusion injury in the liver.^[33–36]^

SARS-CoV-2 can also promote intracellular cytotoxicity to hepatocytes through membrane destruction and mitochondrial dysfunction. The virus might also activate rapamycin signaling, which inhibits autophagy and facilitates viral escape from the immune system of the host cell. Several findings have been proposing that the virus potentially causes damage to bile duct epithelial cells. There is evidence that typical coronavirus particles were identified in the cytoplasm of hepatocytes.^[37]^ Additionally, hepatic mitochondrial swelling and narrowed glycogen granules were observed in the SARS-CoV-2-infected liver cell, which indicated hepatic injury caused by direct cytotoxicity of SARS-CoV-2.^[38]^

Another set of histological findings from liver biopsies of patients with SARS suggested that SARS-CoV-2 may induce apoptosis of hepatocytes.^[39]^ Many infected patients were treated with antipyretic agents containing acetaminophen, a drug that can cause significant liver damage. It is known that acute ingestion of > 7.5 to 10 g of acetaminophen in adults or 150 to 200 mg/kg in children is likely to cause hepatotoxicity. Due to the hepatotoxicity of these drugs, the US Food and Drug Administration Committee has proposed reducing the maximum daily dosage of acetaminophen from 4 to 3 g to minimize drug-related liver damage in SARS-CoV-2 patients.^[40–42]^

## 6. Alteration of some liver biomarkers in SARS-COV-2 induced liver damage

From the above mechanisms of liver damage, it is evident that individuals presenting with COVID-19 have a hepatic injury and abnormal liver function tests. The histopathological changes of the liver might include complete or partial thrombosis of the portal and sinusoidal vessels, portal tract fibrosis, and focally markedly enlarged and fibrotic hepatocytes. Studies have shown an increase in some liver biomarkers, particularly the elevation of ALT and AST levels due to hepatocellular damage. Mild increases in AST and ALT levels have been found in 14% to 53% of severe SARS-CoV-2 infection cases.^[43–45]^ SARS-CoV-2 may damage the biliary tract which increases indirect and total bilirubin, gamma-glutamyl transferase (GGT), and ALP levels. Another study proposed that there are variable levels of increments of GGT, ALP, and ALT observed in 58% to 78% of patients.^[45,46]^

An observational cohort study done in China, in Wuhan, with 2912 COVID-19 patients, found that 1414 patients (48.6%) had abnormal LFTs, with ALT, AST, total bilirubin, ALP, and GGT elevation in 662 (22.7%), 221 (7.6%), 52 (1.8%), 135 (4.6%), and 536 (18.5%), respectively. According to this study, abnormal liver function tests are an independent risk factor for mortality, intensive care unit admission, and mechanical ventilation among COVID-19 patients.^[47]^

## 7. Conclusion

Liver damage is the most serious issue for those with SARS-CoV-2 infection. Any liver damage sustained by COVID-19 patients throughout their illness or treatment is referred to as a COVID-19-associated liver injury. Abnormal liver function tests are typically used to identify liver disease in COVID-19 patients. Moreover, damaged liver cells may reduce the therapeutic effectiveness of antiviral medications. Although the precise cause of liver damage in SARS-CoV-2-infected patients is still unknown, several theories have been put forth, including those involving the direct cytopathogenic effects of the SARS virus, cytokine storms, hypoxia brought on by respiratory failure, and specific medications used to treat COVID-19.

Proinflammatory cytokines and immune cell overactivity result in a cytokine storm that exacerbates further hepatocyte injury. Severe respiratory distress, which may result in tissue hypoxia, is 1 acute consequence of COVID-19 in patients. A liver ischemia injury develops if the liver’s oxygen delivery is impaired. Inadequate oxygen delivery to the liver will result in aberrant lipid accumulation, metabolic abnormalities, and adinosine triphosphate depletion, which kills hepatocyte cells.

Elevated liver enzymes including ALT, AST, GGT, ALP, and Serum total bilirubin levels are some of the laboratory results after SARS-CoV-2 induced liver injury.

In addition, portal tract fibrosis, sinusoidal artery thrombosis, and fibrotic hepatocytes are histological abnormalities in the liver. Even though viral fragments appeared in SARS-CoV-2-infected liver cells, more proof is required.

## Author contributions

**Conceptualization:** Fikadu Seyoum.

**Validation:** Fikadu Seyoum.

**Visualization:** Fikadu Seyoum.

**Writing – original draft:** Fikadu Seyoum.

**Writing – review & editing:** Fikadu Seyoum.
